# Molecular Characterization of Ticks and Tick-Borne Pathogens in Cattle from Khartoum State and East Darfur State, Sudan

**DOI:** 10.3390/pathogens10050580

**Published:** 2021-05-10

**Authors:** Ehab Mossaad, Alex Gaithuma, Yassir O. Mohamed, Keisuke Suganuma, Rika Umemiya-Shirafuji, Yuma Ohari, Bashir Salim, Mingming Liu, Xuenan Xuan

**Affiliations:** 1 Department of Pathology, Parasitology and Microbiology, College of Veterinary Medicine, Sudan University of Science and Technology, Khartoum P.O. Box 204, Sudan; ehabmssd7@gmail.com; 2 National Research Center for Protozoan Diseases, Obihiro University of Agriculture and Veterinary Medicine, Obihiro, Hokkaido 080-8555, Japan; akiariegaithuma@gmail.com (A.G.); k.suganuma@obihiro.ac.jp (K.S.); umemiya@obihiro.ac.jp (R.U.-S.); lmm_2010@hotmail.com (M.L.); 3 Central Veterinary Research Laboratory (CVRL), Soba, Khartoum P.O. Box 8067, Sudan; yassir_mohammed59@hotmail.com; 4 Research Center for Global Agromedicine, Obihiro University of Agriculture and Veterinary Medicine, Obihiro, Hokkaido 080-8555, Japan; 5 Laboratory of Parasitology, Department of Disease Control, Faculty of Veterinary Medicine, Hokkaido University, Sapporo, Hokkaido 060-0818, Japan; y_ohari@vetmed.hokudai.ac.jp; 6 Faculty of Veterinary Medicine, University of Khartoum, Khartoum-North P.O. Box 32, Sudan; bashirsalim@gmail.com

**Keywords:** ticks, tick-borne pathogens, epidemiology, cattle, Khartoum State, East Darfur State, Sudan

## Abstract

Ticks transmit many pathogens with public health and veterinary importance. Despite the wide distribution of tick-borne pathogens in Sudan, the information on the tick–pathogen relationship needs to be updated, particularly using modern molecular techniques. This cross-sectional study, conducted between September and November 2019, used morphology, PCR, and sequencing to confirm the identity of adult cattle ticks (male and female; n = 536) from Khartoum State (n = 417) and East Darfur State (n = 119). Moreover, the presence of *Theileria annulata*, *Babesia bigemina*, *B. bovis*, *Anaplasma marginale*, and *Ehrlichia ruminantium* was detected and confirmed in each tick using species-specific PCR or nested PCR and sequencing. The most economically important tick genera, *Rhipicephalus, Hyalomma*, and *Amblyomma*, were prevalent in the study area, and 13 different tick species were identified. The most prevalent tick species were *Rhipicephalus* *evertsi evertsi* (34.3%) and *Hyalomma anatolicum* (57.3%) in Khartoum State, and *Rhipicephalus annulatus* (27%), *Rhipicephalus decoloratus* (25%), and *Hyalomma rufipes* (29%) in East Darfur State. We detected all five pathogens in both states. To the best of our knowledge, this is the first study to report the presence of *E. ruminantium*, its vector *Amblyomma variegatum*, and *B. bovis* in Khartoum State. Further, this is the first report on most tick and pathogen species identified in East Darfur State. Our findings indicate the migration of some tick and pathogen species beyond their distribution areas in the country, and this consideration is necessary to develop future control strategies.

## 1. Introduction

Ticks (Acari: Ixodoidea) are important ectoparasites infesting livestock and human populations around the globe [1]. Ticks carry and transmit a large number of pathogens, including bacteria, viruses, and protozoa. They are second to mosquitoes in importance as disease vectors, greatly impacting human and animal health [2]. Ticks are voracious blood suckers, causing heavy blood losses leading to anemia, and the injuries caused by their attachment damage hides and predispose animals to secondary bacterial infections that may lead to mastitis. These wounds may also attract myiasis-causing flies. Long-hypostome ticks may induce abscesses due to secondary bacterial infections [3]. Moreover, ticks can cause paralysis [4]. Ticks have a wide range of hosts, with cattle being the favorite for many tick species [5].

Sudan occupies an area of 1.886 million km^2^ in the northeast of Africa and is located at latitude 15°47′9.85″ N and longitude 30°11′58.48″ E. Sudan harbors one of the largest cattle populations in Africa; the cattle population was estimated at approximately 31 million heads, raised in almost all parts of the country [6]. This large cattle population is severely affected, and its productivity is highly hampered by different ticks and tick borne-diseases. During the late 1940s and early 1950s, Hoogstraal recorded 25 tick species in Sudan [4]. Since then, scattered studies on tick distribution, morphological identification, morphological abnormalities, host range, and role in disease transmission were carried out in the country. These studies reported 70 tick species, including *Hyalomma anatolicum*, *H*. *impressum*, *H*. *dromedarii*, *H*. *impeltatum*, *H*. *rufipes*, *H*. *truncatum*, *Rhipicephalus evertsi evertsi*, *R*. *sanguineus*, *R*. *praetextatus*, *R*. *annulatus*, *R*. *decoloratus*, *Amblyomma lepidum*, and *Am*. *variegatum*, as the most prevalent tick species in the country [7,8,9,10,11,12,13,14,15,16]. As mentioned, ticks are very important disease vectors for livestock in Sudan; however, owing to the limited resources to perform molecular-based surveys, very few reports on the molecular identification of ticks, with data on only *R*. *sanguineus*, are available [14]. Moreover, very few studies have identified tick-borne pathogens in ticks in Sudan, such as *Ehrlichia ruminantium* in *Am*. *lepidum* and *Am*. *variegatum* [17,18], *Rickettsia aeschlimannii* and *R. africae* in different *Hyalomma* spp. and *Am*. *lepidum* [14], Crimean-Congo hemorrhagic fever virus in *H*. *impeltatum* [19], and *R. africae* in *Am*. *variegatum* [20]. Sudan consists, on the north-south direction, of desert, a semi-desert and a savannah that exhibits unique flora and fauna, with five identified ecological zones of rainfall [21]. In addition to this, its unique location in Africa, connecting north Africa with sub-Saharan countries, makes Sudan a very important country prone to transboundary animal diseases, including ticks and tick-borne diseases. Moreover, many factors determine the dynamic changes in tick distribution in the country, including animal movement for trade, nomadism, or migration due to civil unrest, which have been discussed in detail by Hassan and Salih [21]. Therefore, intensive and continuous screening for ticks and tick-borne diseases in Sudan is essential. The present study investigated the distribution and species of ticks infesting cattle in Khartoum State, central Sudan, and East Darfur State, western Sudan. We further screened the collected ticks for the presence of the most economically important tick-borne pathogens, including *Thielira annulata*, *Babesia bovis*, *B. bigemina*, *Anaplasma marginale*, and *Ehrlichia ruminantium*.

## 2. Results

### 2.1. Tick Species Identification

A total of 536 ticks were examined in this study. All individual ticks were morphologically identified. We identified three tick genera: *Rhipicephalus*, *Hyalomma*, and *Amblyomma*. The genus *Hyalomma*, with a prevalence of 56.9% (305/536), was found to be the most prevalent, followed by the genus *Rhipicephalus* [40.9% (219/536)] and *Amblyomma* [2.6% (14/536)].

Within the three genera, 13 tick species were morphologically identified, namely, *R. annulatus*, *R. decoloratus*, *R. e. evertsi*, *R. praetextatus*, *R. simpsoni*, *Hyalomma rufipes*, *H. anatolicum*, *H. excavatum*, *H. dromedarii*, *H. impeltatum*, *H. marginatum*, *Am. lepidum*, and *Am. variegatum* (Table 1). In general, within the identified tick species, *H. anatolicum* [44.6% (239/517)] and *R. e. evertsi* [27% (144/536)] were the most prevalent in the study area (Table 1).

A comparison of the prevalence of the different tick species in Khartoum State and East Darfur State revealed that the prevalence of *H. anatolicum* [57.3% (239/417)] and *R. e. evertsi* [34.3% (143/417)] in Khartoum State was significantly higher (*p* < 0.05) than that in East Darfur State, which was calculated to be 0% (0/119) and 0.84% (1/119), respectively (Table 1). In contrast, in East Darfur State, the prevalence of *R. annulatus* [27% (32/119)], *R. decoloratus* [25% (30/119)], *H. rufipes* [29% (34/119)], *H. impeltatum* [7.6% (9/119)], and *Am. lepidum* [8.4% (10/119)] was significantly higher (*p* < 0.05) than that in Khartoum State which [0% (0/417), 0% (0/417), 1.2% (5/417), 0.24% (1/417), and 0% (0/417), respectively] (Table 1). No statistically significant difference (*p* > 0.05) was observed between the prevalence of the remaining tick species in the two states (Table 1).

One to 15 ticks from all tick species morphologically identified were randomly selected for further confirmation using mitochondrial 12S rDNA gene analysis. The lengths of the amplified tick mitochondrial 12S rDNA sequences varied from 306 bp to 349 bp. A BLASTn analysis of the sequences obtained in this study showed an identity ranging from 97.64% to 100% with reference sequences from GenBank. However, matching mitochondrial 12S rDNA reference sequences for *R. simpsoni* are not available in the GenBank. All sequencing data are provided in Supplementary Table S1.

### 2.2. Pathogens Detected in the Ticks and Infection Rates

All tick DNA samples (536) were examined by PCR for the presence of five pathogens: *Theileria* *annulata*, *Babesia bigemina*, *Babesia bovis*, *Anaplasma marginale*, and *Ehrlichia ruminantium*. The most frequently observed pathogens in the tick samples were *T. annulata* [37.3% (200/536)] and *B. bigemina* [18.7% (100/536)], followed by *A. marginale* [10.6% (57/536)], *E. ruminantium* [10.07% (54/536)], and *B. bovis* [5.6% (30/536)] (Table 2). In Khartoum State the prevalence of *T. annulata* [42.7% (178/417)], *A. marginale* [12.5% (52/417)], and *E. ruminantium* [12.2% (51/417)] was significantly higher (*p* < 0.05) than that in East Darfur State [18.5% (22/119), 4.2% (5/119), and 2.5% (3/119), respectively]. In contrast, the prevalence of *B. bigemina* [31.1% (37/119)] in East Darfur State was significantly higher (*p* < 0.05) than that recorded in Khartoum State [15.1% (63/417)]. The prevalence of *B. bovis* in ticks did not differ to a statistically significant extent between the two states (Table 2).

### 2.3. Distribution of Tick-Borne Pathogens within Different Tick Species

In general, out of 536 tick samples examined, 339 (63%), representing 11 tick species, were positive for at least one of the five pathogens detected, while only two tick species, *H. dromedarii* and *R. simpsoni*, were negative for all pathogens. On the other hand, *R. e. evertsi*, *H. anatolicum*, and *H. marginatum* were found to harbor all the five pathogens (Table 3).

*Theileria annulata*, the most prevalent pathogen in the study samples, with a prevalence of 37.3% (200/536) (Table 2), was found mainly in two tick species, *R. e. evertsi* [27.5% (55/200)] and *H. anatolicum* [56.5% (113/200)] (Table 3). *B. bigemina*, the second most prevalent pathogen, with a prevalence of 18.7% (100/536) (Table 2), was found mainly in four tick species, *H. anatolicum* [44% (44/100)], *R. decoloratus* [18% (18/100)], *R. e. evertsi* [15% (15/100)], and *R. annulatus* [11% (11/100)] (Table 3). Other relatively less prevalent pathogens, including *B. bovis*, *A. marginale*, and *E. ruminantium*, were found harbored in *R. e. evertsi* with a prevalence of 23.3% (7/30), 35.1% (20/57), and 24.1% (13/54), respectively, and in *H. anatolicum* with a prevalence of 43.3% (13/30), 49.1% (28/57), and 55.5% (30/54), respectively (Table 3).

### 2.4. Comparative Sequence Analyses of tams-1, ama1, msp4, spb4, and pCS20 Genes

The percentage of nucleotide identity of all four *T. annulata tams-1* gene sequences (LC611432–LC611435) ranged from 94.7% to 95.4% among themselves. These sequences showed a 99.6% and 97% nucleotide sequence identity with sequences from previous studies conducted in Mauritania (AF214823) and India (MH277619). Further, the percentage of identity of all five sequences of *B. bigemina ama1* gene (LC611412–LC611416) ranged from 98.1% to 99.05% among themselves. These sequences showed a 99% nucleotide sequence identity with sequences from Egypt (AB917262) and South Africa (KF626596). On the other hand, the percentage of nucleotide identity of all seven *B. bovis spb4* gene sequences (LC611417–LC611423) ranged from 97.9% to 99.7% among themselves. These sequences showed a 99.6% and 95% nucleotide identity with sequences from previous studies conducted in Indonesia (KY484532) and South Africa (KF626635). Furthermore, the percentage of nucleotide identity of nine *A. marginale msp4* gene sequences (LC611403- LC611411) ranged from 99.4% to 100% among themselves. These sequences shared a 100% nucleotide sequence identity with a previously reported sequence from Nigeria (EU106082). Additionally, the percentage of nucleotide identity of seven *E. ruminantium* pCS20 gene sequences (LC611424–LC611430) ranged from 98.9% to 99.8%. However, these sequences shared a 100% and 99.3% nucleotide sequence identity with previously reported sequences from South Africa (DQ631925) and Sudan (AB218277).

### 2.5. Phylogenetic Analysis

**Figure 1:**
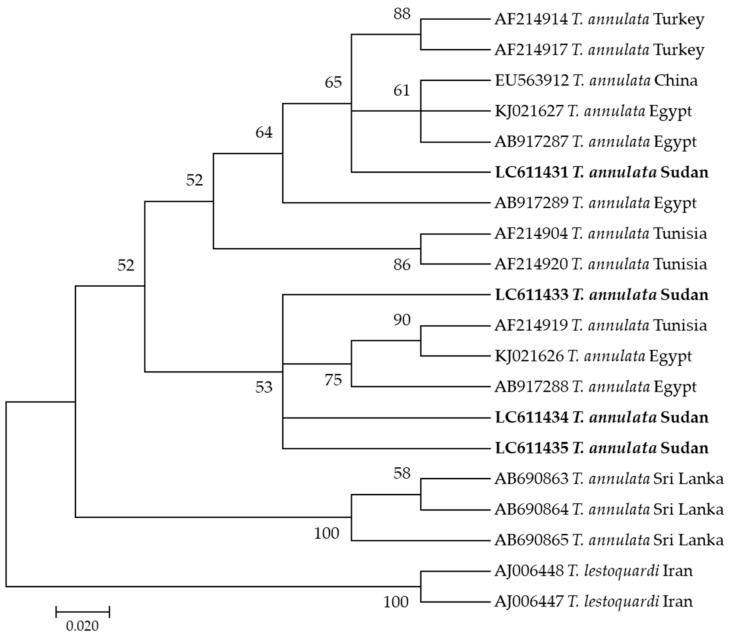
Phylogenetic analysis of *Theileria annulata* identified in this study based on *Tams-1* gene sequences using the maximum likelihood method. The number at the nodes represents the percentage occurrence of clade in a 1000 bootstrap replication of data. The best model was Tamura-Nei Model 92 with Gamma distribution. Sequences from this study are shown in bold font. The tree was constructed using the MEGA version X software program.

In this study, phylogenetic trees of *T. annulata*, *B. bigemina*, *B. bovis*, *A. marginale*, and *E. ruminantium* were constructed based on *tams-1*, *ama1*, *spb4*, *msp4*, and *pCS20* genes, respectively, using sequences from the NCBI GenBank. Four sequences (LC611432–LC611435) of *T. annulata* were analyzed. LC611431 clustered on one clade of the phylogenetic tree with sequences AB917287 and KJ021627 from Egypt, while the other three sequences (LC611433, LC611434, and LC611435) clustered together with AF214919 from Tunisia and KJ021626 and AB917288 from Egypt (Figure 1).

**Figure 2:**
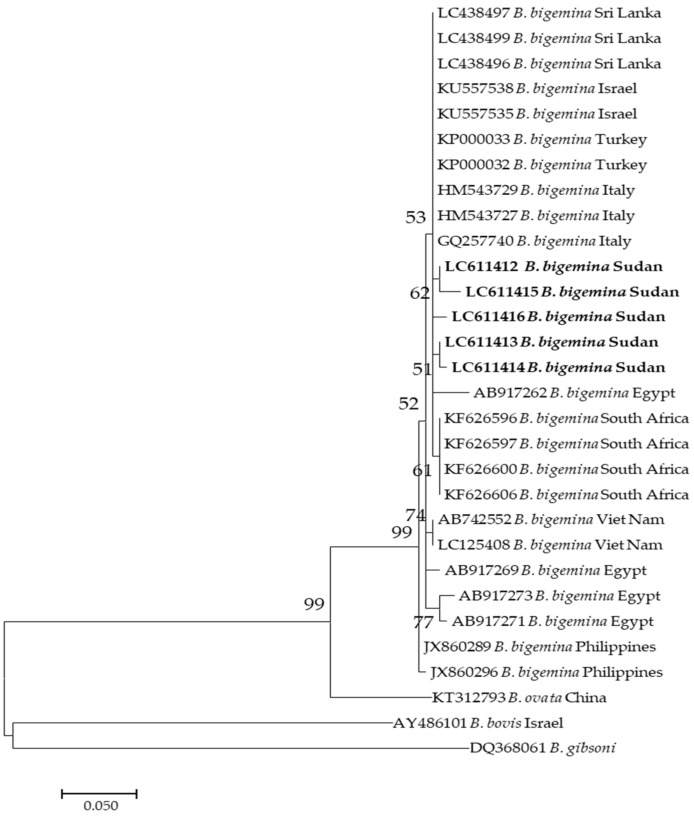
Phylogenetic analysis of *Babesia bigemina* identified in this study based on *ama1* gene sequences using the maximum likelihood method. The number at the nodes represents the percentage occurrence of clade in a 1000 bootstrap replication of data. The best model was the Kimura 2-parameter model. Sequences from this study are shown in bold font. The tree was constructed using the MEGA version X software program.

On the other hand, five sequences (LC611412–LC611416) of *B. bigemina* used in this study appeared in the same clade, that also involved sequences KF626596, KF626597, and KF626600 from South Africa as well as AB17262 from Egypt (Figure 2).

**Figure 3:**
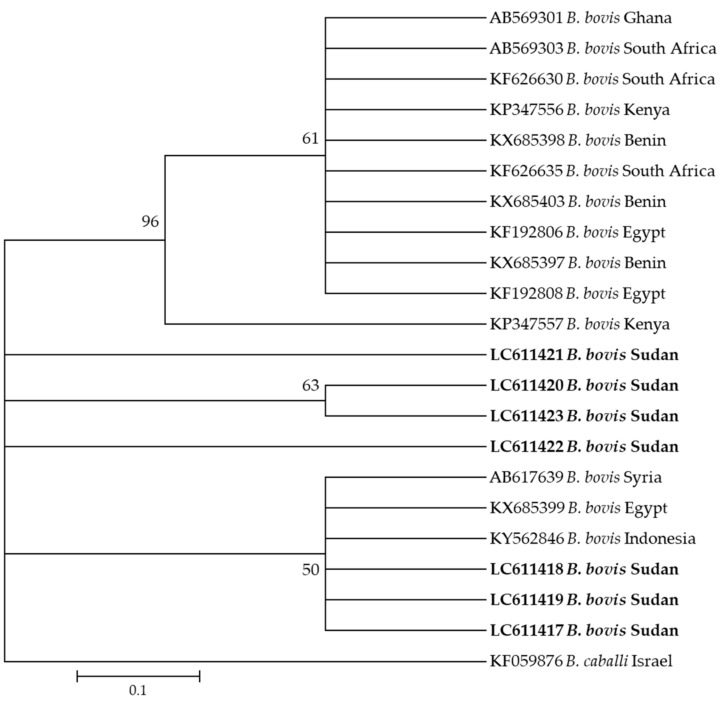
Phylogenetic analysis of *Babesia bovis* identified in this study based on *spb4* gene sequences using the maximum likelihood method. The number at the nodes represents the percentage occurrence of clade in a 1000 bootstrap replication of data. The best model was the Kimura 2-parameter model with invariant sites. Sequences from this study are shown in bold font. The tree was constructed using the MEGA version X software program.

Moreover, seven sequences (LC611417–LC611423) of *B. bovis* generated in this study clustered together in the same clade with sequences KY484532 from Indonesia, KX685399 from Syria, and KX685399 from Egypt (Figure 3).

**Figure 4:**
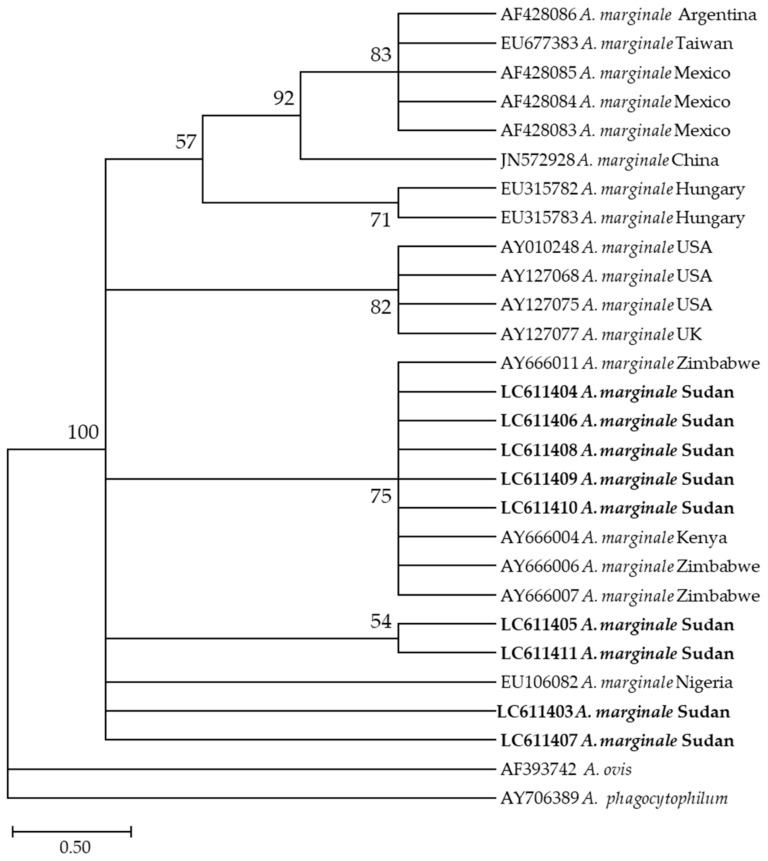
Phylogenetic analysis of *Anaplasma marginale* identified in this study based on *msp4* gene sequences using the maximum likelihood method. The number at the nodes represents the percentage occurrence of clade in a 1000 bootstrap replication of data. The best model was the Tamura 2-parameter model with invariant sites. Sequences from this study are shown in bold font. The tree was constructed using the MEGA version X software program.

Furthermore, the phylogenetic analysis revealed that the nine *A. marginale* sequences generated in this study (LC611403–LC611411) were in the same clade as sequences from Zimbabwe (AY666007, AY666011, and AY666006), from Kenya (AY666004), and from Nigeria (EU106082) (Figure 4).

**Figure 5:**
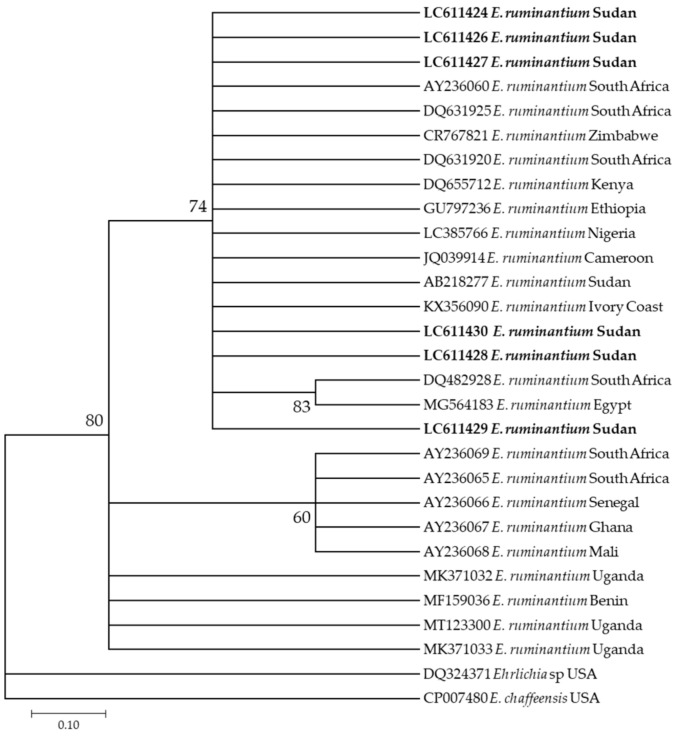
Phylogenetic analysis of *Ehrlichia ruminantium* identified in this study based on *pCS20* gene sequences using the maximum likelihood method. The number at the nodes represents the percentage occurrence of clade in 1000 a bootstrap replication of data. The best model was Tamura-Nei Model 92. Sequences from this study are shown in bold font. The tree was constructed using the MEGA version X software program.

Additionally, six *E. ruminantium* sequences (LC611424–LC611430) used in this study appeared in the same clade along with different sequences from different countries in Africa, such as those from South Africa (DQ631925, DQ631920, and AY236069) and a previously reported sequence from Sudan (AB218277) (Figure 5).

## 3. Discussion

Tick infestations are common in Africa, including Sudan, where tick fauna comprises over 70 species prevalent in diverse ecological zones [21]. In this study, ticks were collected from cattle in two states—Khartoum State, in central Sudan, and East Darfur State, in western Sudan. The ticks were examined using morphology and molecular techniques (PCR, sequencing, and phylogenetics) to identify the species as well as to detect the microorganisms they carried. We morphologically identified 13 tick species of the three most economically important genera, namely, *Rhipicephalus*, *Hyalomma*, and *Amblyomma*, and confirmed their identity using molecular techniques. Within the genus *Rhipicephalus*, five different species were identified. *R. annulatus* and *R. decoloratus*, the main vectors of babesiosis [22], were detected only in East Darfur State. This is consistent with previous studies [4,16,23,24] that confirmed the presence of these two tick species in southern parts of the county, extending as far north as Wad Medani (14° 25′ N, 32° 75′ E), which lies to the south of Khartoum State, where these two ticks were not identified in this study. Hassan and Salih attributed the presence of these two ticks in the southern parts of the country, including East Darfur State, to humidity; Khartoum State is a dry area [21]. Interestingly, this is the first study to identify *R. simpsoni* tick species (the greater cane rat tick), which is considered native to the Republic of South Sudan, in Khartoum State [4,25,26]. The presence of *R. simpsoni* in Khartoum State, hundreds of kilometers north of the Republic of South Sudan, indicates that ticks in general are highly dynamic and can move with the animals migrating for different reasons, such as nomadism or civil unrest, especially after the separation of Sudan and the Republic of South Sudan in 2011, as discussed by Hassan and Salih [21]. We detected a significantly higher prevalence of *R. e. evertsi* in Khartoum Sate. With a prevalence of 34.3%, *R. e. evertsi* was the second most abundant tick species in Khartoum State after *H. anatolicum*. However, only one tick belonging to *R. e. evertsi* was recorded in East Darfur State, while all *R. praetextatus* ticks identified in this study were from Khartoum State. This data on *R. e. evertsi* and *R. praetextatus* is consistent with their distribution recorded in previous studies [27,28,29]. Within the genus *Hyalomma*, the prevalence of *H. anatolicum*, the most important tick species in Sudan and the main vector for *T. annulata* in the country [21], was found to be significantly higher in Khartoum State. This finding is consistent with previous studies [8,30]. *H. anatolicum* is a xerophilic species thriving in semi-desert conditions in northern Sudan, including Khartoum Sate [10]. Although in this study we did not detect *H. anatolicum* in East Darfur State, Abdalla and Hassan have identified it in Nyala, in South Darfur State [31], which borders East Darfur Sate. The absence of this tick species in East Darfur State in our study confirms that *H. anatolicum* is established in Nyala, South Darfur Sate, but has not yet spread to other localities of Darfur, as discussed previously [21]. We also reported a significantly higher prevalence of *H. impeltatum* and *H. rufipes* in East Darfur State that is consistent with the findings of previous studies [29,32] that reported their prevalence in South Darfur Sate, bordering East Darfur Sate. Two ticks of the genus *Amblyomma* were detected in this study. *Am. lepidum*, which is classified as a dry region tick abundant in the eastern part of Sudan [15,33], was detected in far western Sudan in East Darfur. Although this tick was previously reported in South Darfur State [31], the present study is the first report in East Darfur State. The presence of *Am. lepidum* in East Darfur State is alarming because it suggests the possible spread of this species, that was previously confined to eastern parts of the African continent [33], to West African countries in the near future. This would aggravate and complicate the heartwater incidence in West African countries, as discussed by Hassan and Salih [21]. On the other hand, within *Amblyomma*, only two female *Am. variegatum* ticks were detected in Khartoum State. The occurrence of *Am. variegatum* in Khartoum State is surprising because this tick species has not been previously identified in the area; however, it explains the presence of *E. ruminantium*, detected in this study for the first time in Khartoum State.

We investigated five main tick-borne pathogens, *T. annulata*, *B. bigemina*, *B. bovis*, *A. marginale*, and *E. ruminantium*, in Sudan. All pathogens detected in this study were further confirmed using sequencing and phylogenetic analyses by clustering them with those in the GenBank. In Khartoum State, endemic for *T. annulata* and *H. anatolicum* [34], the parasite was detected in 42.7% (178/417) of the ticks, and its main vector, *H. anatolicum*, showed a 57.3% (239/417) prevalence. Interestingly, although *H. anatolicum* was not detected in East Darfur State in this study, we detected *T. annulata* for the first time in this area, in 18.5% (22/119) of the ticks belonging to different species of the genus *Hyalomma*. These data indicate the presence of alternative vectors for *T. annulata* in East Darfur State, such as *H. rufipes*, *H. impeltatum*, and *H. marginatum*, that were previously shown to transmit the parasite [34,35,36,37]. Although *T. annulata* was not detected in *H. dromedarii* in this study, it can act as an alternative vector [35]. We identified two *Babesia* parasites, *B. bigemina* and *B. bovis*, in the present study. In East Darfur State, both these parasites and their classical vectors, *R. annulatus* and *R. decoloratus* [22], were found. Similarly, we confirmed the presence of both *B. bigemina* and *B. bovis* in ticks from Khartoum State. Supporting this, *B. bigemina* was previously detected and microscopically identified in two cows in Khartoum Sate [38]. Notably, *R. annulatus* and *R. decoloratus* were not found in Khartoum State in this study. This finding should alert the veterinary authorities to the fact that these two *Babesia* parasites are prevalent, and control measures should be implemented, especially considering the high prevalence of *R. e. evertsi* (34.3%) in Khartoum Sate, which can transmit these parasites as an alternative vector [22]. This study is the first to confirm the presence of *E. ruminantium* and its vector *Am. variegatum* in Khartoum State. Our finding indicates that the bacterium and its vector are well established in this area and may have been overlooked in previous studies. This is cause for alarm, and the authorities should begin an organized screening of the clinical cases to develop better control strategies. More studies investigating the biological aspects of *Am. variegatum* and its presence in Khartoum State, which was considered free of this tick species [4,25], are required. In the present study, the prevalence of *E. ruminantium* in East Darfur State was consistent with that of its vector, *Am. lepidum*. It is worth mentioning that this is the first report on the presence of both the pathogen and its vector in East Darfur State, although they were previously detected in South Darfur State [18,31]. In this study, the prevalence of *A. marginale* was confirmed in ticks from Khartoum State and East Darfur State. Like most of the pathogens investigated in this study, this is the first report on the prevalence of *A. marginale* in East Darfur State. *A. marginale* was previously reported in Khartoum State [38,39]. Further, *Anaplasma marginale* was proven to be transmitted by *H. rufipes*, *R. annulatus*, *R. decoloratus*, and *R. e. evertsi* [35]. All these tick species were identified in the two states in this study except for *R. annulatus* and *R. decoloratus*, that were only identified in East Darfur State.

Although all ticks assessed in this study were feeding ticks collected from the animals, and the pathogen source may be the infested animal itself, all identified tick species harboring the respective pathogens could be considered as potential vectors for these pathogens. This study provides basic information on the prevalence of the most economically important ticks and tick-borne pathogens in Khartoum State and East Darfur State. Our findings provide a better understanding of the tick–pathogen relationship in Sudan. Studies on the prevalence and distribution of ticks and tick-borne pathogens in Sudan are highly important to develop better control strategies.

## 4. Materials and Methods

### 4.1. Study Area

**Figure 6:**
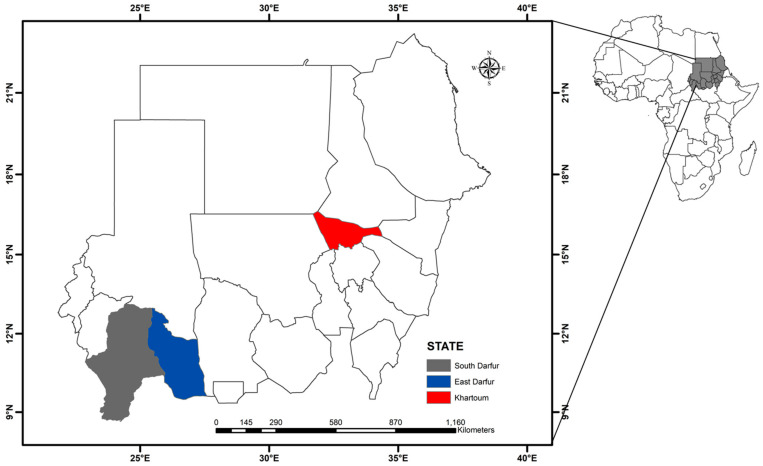
A map of Sudan. Map created using the ArcMap 10.1 software program (Esri, Redlands, CA, USA).

This cross-sectional study was carried out between September and November 2019 in Khartoum State, central Sudan (longitude 31.5° to 34° E and latitude 15° to 16° N), and East Darfur State, western Sudan (longitude 26° to 30° E and latitude 11° to 10.01° N) (Figure 6). Although Khartoum State is the smallest state in the country based on area (22,142 km^2^), it is considered a big market, and livestock is transported from different parts of the country to meet the consumer demand of meat [40]. Moreover, 17 dairy camps were established around Khartoum State, under the supervision of the Ministry of Agriculture, Animal Resources, and Irrigation of Khartoum State, under an intensive traditional farming system, to meet approximately 80% of the milk requirement of the population [41]. In contrast, in East Darfur State, livestock is owned by the traditional sector, mostly made up of nomads who migrate with their animals as far as beyond latitude 8° N in the south and 14° N in the north, in search for pastures throughout the year [21]. Considering the differences in their geographic location and animal husbandry practices, we selected these two states for tick screening in this study.

### 4.2. Tick Samples

A total of 536 adult feeding ticks [male (n = 328) and female (n = 208)] were collected from 153 infested cattle (three to five ticks from each animal) in Khartoum State (n = 417) and East Darfur State (n = 119). The tick sampling was conducted with the consent of the livestock owners, and care was taken to minimize animal discomfort. All procedures of tick sampling and sample processing were performed in accordance with the ethical guidelines of the Obihiro University of Agriculture and Veterinary Medicine (approval No: 2019020 and 200049). Collected ticks were isolated in 1.5 mL tubes containing 70% ethanol, labeled with the location and date of collection, and stored at room temperature until processing. Prior to DNA extraction, identification and morphological classification of the tick species were carried out using a binocular microscope (Olympus SZX16, Tokyo, Japan) with previously established standard taxonomic keys [4,35].

### 4.3. DNA Extraction

Each tick was washed in three 70% ethanol baths, rinsed in double distilled water, air dried, and collected in sterile 1.5 mL tubes. The tick-containing tubes were plunged in liquid nitrogen for 3–5 min, and the frozen ticks were crushed using sterile pellet mixers. The tissues of crushed ticks were transferred aseptically to sterile 1.5 mL tubes and used for DNA extraction. DNA was extracted using NucleoSpin^®^ Tissue Mini kit for DNA from cells and tissue (Macherey-Nagel, Dueren, Germany) according to the manufacturer’s protocol. Extracted DNA was stored at −30 °C until molecular analysis.

### 4.4. Molecular Characterization of Ticks

To confirm the species, tick DNA was amplified by PCR, and an approximately 360-bp fragment of tick mitochondrial 12S rDNA was sequenced. The amplification was performed with forward (F1; 5′-AAACTAGGATTAGATACCCT-3′) and reverse (R1; 5′-AATGAGAGCGACGGGCGATGT-3′) primers [42] under the following cycling conditions: initial denaturation at 98 °C for 2 min, 40 cycles of denaturation at 98 °C for 10 s, annealing at 55 °C for 15 s, and extension at 68 °C for 45 s. Each 10 µL PCR reaction method included 5 µL of 2× MightyAmp Buffer Ver.3, 1 µL of 10× Additive for High Specificity, 0.2 µL of MightyAmp DNA Polymerase Ver. 3 (Takara, Shiga, Japan), 0.5 µL each of 10 mM forward and reverse primers, 1.8 µL of double-distilled water, and 1 µL of the tick DNA template. The DNA of *Amblyomma variegatum* ticks collected in Benin [43] and distilled water were used as positive and negative controls, respectively. PCRs were conducted on a Veriti^™^ Thermal cycler (Thermo Fisher Scientific Inc., Waltham, MA, USA). The PCR products were electrophoresed on 1.5% agarose gels that were stained with ethidium bromide and visualized under UV light. Then, all amplicons were extracted from the gels using a QIAquick Gel Extraction Kit (QIAGEN, Hilden, Germany) and sequenced.

### 4.5. Detection and Characterization of Tick-Borne Pathogens

To detect the presence of *T. annulata*, *B. bigemina*, *B. bovis*, *A. marginale*, and *E. ruminantium* in the extracted DNA, individual tick DNA was used as template to amplify the *T. annulata* merozoite surface antigen (*tams-1*) [44], *B. bigemina* apical membrane antigen-1 (*ama1*) [44], *B. bovis* spherical body protein-4 (*sbp4*) [45], *A. marginale* major surface protein 4 (*msp4*) [46], and *E. ruminantium* pCS20 region [47]. The primer sequences used in all PCR assays are listed in Table 4. PCR cycling conditions for all pathogen assays were the same as documented in respective referenced publications, with some modifications. Briefly, the cycling conditions were as follows: initial denaturation at 98 °C for 2 min; 40 cycles of denaturation at 98 °C for 10 s, annealing at varying temperatures (indicated in Table 4) for 15 s, and extension at 68 °C for 45 s. The reaction mixtures included MightyAmp^TM^ DNA Polymerase Ver. 3 (Takara, Shiga, Japan), and the amplified templates were observed as described in 4.4. Amplicons were purified using a QIAquick Gel Extraction Kit and used for sequencing.

### 4.6. Sequencing Analysis

The selected gel-extracted PCR amplicons were sequenced bi-directionally using the same PCR primers. Approximately 300 ng/µL of purified DNA was used for sequencing the PCR amplicons using a Big Dye Terminator kit. The PCR product was ethanol-precipitated and dissolved in 20 µL of a Hi-Di formamide solution prior to DNA sequencing. The sequencing procedure consisted of 30 cycles of denaturation at 96 °C for 1 min, annealing at 50 °C for 5 s, and extension at 60 °C for 2 min. The gene sequence was analyzed using an ABI Prism 3100 Genetic Analyzer (Applied Biosystems, Carlsbad, CA, USA). The forward and reverse raw sequences were processed using sangeranalyseR [48] to generate consensus sequences. Low quality sequences were excluded and cleaned manually.

### 4.7. BLASTn Analysis, Sequence Alignment, and Phylogenetic Analyses

Pairwise distances among the sequences were evaluated using MEGA version X [49], and the percentage identity was determined by the Basic Local Alignment Search Tool (BLASTn) in NCBI GenBank. Reference sequences were downloaded from NCBI, and the Simulate PCR tool was used to extract amplicons with respective primers. The reference extracts and sequenced products were aligned using MAFFT online version 7 (https://mafft.cbrc.jp/alignment/software/(accessed date 15 February 2021)). The alignments were imported to MEGA version X, and modeling was performed to determine the substitution model for each alignment. The phylogenetic analysis was performed by Maximum Likelihood using the respective substitution models and 1000 bootstraps. The sequences generated in this study were deposited in the GenBank database under the accession numbers (LC612433–LC612524 for different tick species), (LC611403–LC611411 for *A. marginale*), (LC611412–LC611416 for *B. bigemnia*), (LC611417–LC611423 for *B. bovis*), (LC611424–LC611430 for *E. ruminantium*), and (LC611431–LC611435 for *T. annulata*).

### 4.8. Statistical Analysis

The prevalence (P) of different ticks and tick-borne pathogens was calculated using the following formula: P (%) = number of positive samples/total number of samples × 100. A Chi-squared test and a Fisher’s exact test were used to investigate the differences in the prevalence with different variables using GraphPad (GraphPad Software Inc., San Diago, CA, USA). *p-*values < 0.05 were considered to indicate statistically significant differences.

## 5. Conclusions

We documented the prevalence of 13 different tick species belonging the most economically important tick genera, *Rhipicephalus*, *Hyalomma*, and *Amblyomma*, in two states in Sudan, namely, Khartoum Sate and East Darfur State. Moreover, we documented five pathogens, namely, *T. annulata*, *B. bigemina*, *A. marginale*, *B. bovis*, and *E. ruminantium*, in the collected ticks. We confirmed the identity of all ticks and pathogens using molecular techniques and suggested the potential roles of different tick species in the transmission of the respective pathogens. To the best of our knowledge, this is the first study to detect the presence of *E. ruminantium*, its vector *Am. variegatum*, and *B. bovis* in Khartoum State. Moreover, as very few studies have been carried out in East Darfur State, this is the first report on the most identified tick and pathogen species in East Darfur State. These findings should alert the veterinary authorities to the need for an effective control strategy based on the distribution of ticks and pathogens in the study area as well as throughout the country. Our findings provide insights that could be used for the improvement of diagnostic protocols and the development of better treatment and control strategies of the detected pathogens. Further studies are required to identify the exact role of each tick species in the transmission of the corresponding pathogens. Further studies are anticipated to confirm the establishment of different tick species, especially in Khartoum State.

## Figures and Tables

**Table 1:** Prevalence of different tick species in Khartoum State and East Darfur State.

Tick Species	Khartoum State	East Darfur State	Total
*R. annulatus*	0% (0/417)	27% (32/119) *	6% (32/536)
*R. decoloratus*	0% (0/417)	25% (30/119) *	5.6% (30/536)
*R. e. evertsi*	34.3% (143/417) *	0.84% (1/119)	27% (144/536)
*R. praetextatus*	2.6% (11/417)	0% (0/119)	2% (11/536)
*R. simpsoni*	0.5% (2/417)	0% (0/119)	0.4% (2/536)
*H. rufipes*	1.2% (5/417)	29% (34/119) *	7.3% (39/536)
*H. anatolicum*	57.3% (239/417) *	0% (0/119)	44.6% (239/517)
*H. excavatum*	2.4% (10/417)	0% (0/119)	1.9% (10/536)
*H. dromedarii*	0% (0/417)	0.84% (1/119)	0.2% (1/536)
*H. impeltatum*	0.24% (1/417)	7.6% (9/119) *	1.9% (10/536)
*H. marginatum*	0.96% (4/417)	1.7% (2/119)	1% (6/536)
*Am. lepidum*	0% (0/417)	8.4% (10/119) *	1.9% (10/536)
*Am. variegatum*	0.5% (2/417)	0% (0/119)	0.4% (2/536)
Total	417	119	536

R.: *Rhipicephalus*, Am.: *Amblyomma*, H.: *Hyalomma.* * *p* < 0.05 with Chi-Square test or Fisher Exact Probability Test.

**Table 2:** Prevalence of different tick-borne pathogens in ticks in Khartoum State and East Darfur State.

PCR	*T. annulate* *tams-1*	*B. bigemina ama1*	*A. marginale msp4*	*B. bovis* *spb4*	*E. ruminantium* *pCS20*
Khartoum State	42.7% (178/417) *	15.1% (63/417)	12.5% (52/417) *	5.5% (23/417)	12.2% (51/417) *
East Darfur State	18.5% (22/119)	31.1% (37/119) *	4.2% (5/119)	5.8% (7/119)	2.5% (3/119)
Total	37.3% (200/536)	18.7% (100/536)	10.6% (57/536)	5.6% (30/536)	10.07% (54/536)

* *p* < 0.05 with Chi-Square test and Fisher Exact Probability Test for the prevalence of *E. ruminantium.*

**Table 3:** Distribution of tick-borne pathogens within different tick species.

Tick Species	*T. annulata*	*B. bigemina*	*B. bovis*	*A. marginale*	*E. ruminantium*
*R. annulatus*	0% (0/200)	11% (11/100)	3.3% (1/30)	1.8% (1/57)	0% (0/54)
*R. decoloratus*	0% (0/200)	18% (18/100)	0% (0/30)	5.3% (3/57)	0% (0/54)
*R. e. evertsi*	27.5% (55/200)	15% (15/100)	23.3% (7/30)	35.1% (20/57)	24.1% (13/54)
*R. praetextatus*	2% (4/200)	1% (1/100)	3.3% (1/30)	1.8% (1/57)	5.6% (3/54)
*R. simpsoni* *	0% (0/200)	0% (0/100)	0% (0/30)	0% (0/57)	0% (0/54)
*H. rufipes*	3.5% (7/200)	2% (2/100)	10% (3/30)	1.8% (1/57)	5.6% (3/54)
*H. anatolicum*	56.5% (113/200)	44% (44/100)	43.3% (13/30)	49.1% (28/57)	55.5% (30/54)
*H. excavatum*	4% (4/200)	4% (4/100)	0% (0/30)	0% (0/57)	5.5% (3/54)
*H. dromedarii* *	0% (0/200)	0% (0/100)	0% (0/30)	0% (0/57)	0% (0/54)
*H. impeltatum*	3% (6/200)	2% (2/100)	10% (3/30)	0% (0/57)	0% (0/54)
*H. marginatum*	1.5% (3/200)	1% (1/100)	6.6% (2/30)	3.5% (2/57)	1.9% (1/54)
*Am. lepidum*	4% (8/200)	2% (2/100)	0% (0/30)	0% (0/57)	1.9% (1/54)
*Am. variegatum*	0% (0/200)	0% (0/100)	0% (0/30)	1.8% (1/57)	0% (0/54)
*Total*	200	100	30	57	54

R.: *Rhipicephalus*, Am.: *Amblyomma*, H.: *Hyalomma.* * No tick-borne pathogen detected.

**Table 4:** Primer sequences used in this study.

Pathogen Target Gene	Assays	Oligonucleotide Sequences (5′→3′)	Annealing Temperature	Product Size (bp)	References
*T. annulata tams-1*	PCR	ATGCTGCAAATGAGGAT	56 °C	768	[44]
		GGACTGATGAGAAGACGATGAG			
*B. bigemina ama1*	PCR	TACTGTGACGAGGACGGATC	62 °C	211	[44]
		CCTCAAAAGCAGATTCGAGT			
*B. bovis sbp4*	PCR	AGTTGTTGGAGGAGGCTAAT	58 °C	907	[45]
		TCCTTCTCGGCGTCCTTTTC			
	nPCR	GAAATCCCTGTTCCAGAG	58 °C	503	[45]
		TCGTTGATAACACTGCAA			
*A. marginale msp4*	PCR	CTGAAGGGGGAGTAATGGG	60 °C	344	[46]
		GGTAATAGCTGCCAGAGATTCC			
*E. ruminantium pCS20*	PCR	CTTGATGGAGGATTAAAAGCA	60 °C	279	[47]
		GTAATGTTTCATGTGAATTGATCC			

## Data Availability

Data is contained within the article and Supplementary Materials.

## Supplementary Materials

The following are available online at https://www.mdpi.com/article/10.3390/pathogens10050580/s1, Table S1: Results of tick species identification based on mitochondrial 12S rDNA gene partial sequence.
