# Supplementary material for: Molecular Characterization of Ticks and Tick-Borne Pathogens in Cattle from Khartoum State and East Darfur State, Sudan
Source: Pathogens. 2021 May 10;10(5):580. doi: 10.3390/pathogens10050580 (PMC8151415; doi:10.3390/pathogens10050580)
Supplement: Supplementary file 1 [file pathogens-10-00580-s001.zip › pathogens-1183431-supplementary.pdf]

Table S1. Results of tick species identification based on mitochondrial 12S rDNA gene partial sequence.

Tick 12S rDNA sequences		Blastn match			
Accession number	Length (bp)	Identity (%)	Accession number	Source	Tick species
LC612490	345	100%	EU921773	Egypt	*Rhipicephalus annulatus*
LC612491	345	100%	EU921773	Egypt	*Rhipicephalus annulatus*
LC612492	345	100%	EU921773	Egypt	*Rhipicephalus annulatus*
LC612493	345	100%	EU921773	Egypt	*Rhipicephalus annulatus*
LC612494	345	99.71%	EU921773	Egypt	*Rhipicephalus annulatus*
LC612495	345	100%	EU921773	Egypt	*Rhipicephalus annulatus*
LC612496	345	100%	EU921773	Egypt	*Rhipicephalus annulatus*
LC612501	340	100%	KY676834	Burkina Faso	*Rhipicephalus decoloratus*
LC612502	340	100%	KY676834	Burkina Faso	*Rhipicephalus decoloratus*
LC612503	340	100%	KY676834	Burkina Faso	*Rhipicephalus decoloratus*
LC612504	340	100%	KY676834	Burkina Faso	*Rhipicephalus decoloratus*
LC612505	340	100%	KY676834	Burkina Faso	*Rhipicephalus decoloratus*
LC612506	340	100%	KY676834	Burkina Faso	*Rhipicephalus decoloratus*
LC612507	340	100%	KF569940	Mali	*Rhipicephalus decoloratus*
LC612508	340	100%	AF150044	Zimbabwe	*Rhipicephalus decoloratus*
LC612476	344	99.41%	MN315381	Senegal	*Rhipicephalus evertsi evertsi*
LC612477	344	99.41%	MN315381	Senegal	*Rhipicephalus evertsi evertsi*
LC612478	344	99.41%	MN315381	Senegal	*Rhipicephalus evertsi evertsi*
LC612479	344	99.41%	MN315381	Senegal	*Rhipicephalus evertsi evertsi*
LC612480	344	99.41%	MN315381	Senegal	*Rhipicephalus evertsi evertsi*
LC612481	344	99.41%	MN315381	Senegal	*Rhipicephalus evertsi evertsi*
LC612483	344	99.12%	MN315381	Senegal	*Rhipicephalus evertsi evertsi*
LC612517	339	97.94%	KX377409	Ethiopia	*Rhipicephalus praetextatus*
LC612518	339	97.94%	KX377409	Ethiopia	*Rhipicephalus praetextatus*
LC612519	339	97.94%	KX377409	Ethiopia	*Rhipicephalus praetextatus*
LC612520	339	97.64%	KX377409	Ethiopia	*Rhipicephalus praetextatus*
LC612523	339	NA	NA	NA	*Rhipicephalus simpsoni*
LC612524	339	NA	NA	NA	*Rhipicephalus simpsoni*
LC612470	341	100%	MH094502	Saudi Arabia	*Hyalomma rufipes*
LC612471	341	99.40%	MH094502	Saudi Arabia	*Hyalomma rufipes*
LC612472	340	98.11%	AF031856	Zimbabwe	*Hyalomma rufipes*
LC612473	341	99.70%	MH094502	Saudi Arabia	*Hyalomma rufipes*
LC612474	341	100%	MH094502	Saudi Arabia	*Hyalomma rufipes*
LC612475	341	100%	MH094502	Saudi Arabia	*Hyalomma rufipes*
LC612442	335	99.11%	KF583616	China	*Hyalomma anatolicum*
LC612443	335	99.11%	KF583616	China	*Hyalomma anatolicum*
LC612444	335	99.11%	KF583616	China	*Hyalomma anatolicum*
LC612445	335	99.11%	KF583616	China	*Hyalomma anatolicum*

LC612446	335	99.11%	KF583616	China	*Hyalomma anatolicum*
LC612447	335	99.11%	KF583616	China	*Hyalomma anatolicum*
LC612448	335	99.11%	KF583616	China	*Hyalomma anatolicum*
LC612449	335	99.11%	KF583616	China	*Hyalomma anatolicum*
LC612450	335	99.11%	KF583616	China	*Hyalomma anatolicum*
LC612451	335	99.11%	KF583616	China	*Hyalomma anatolicum*
LC612452	336	98.22%	KF583616	China	*Hyalomma anatolicum*
LC612453	325	98.15%	KT391040	Israel	*Hyalomma excavatum*
LC612454	325	98.15%	KT391040	Israel	*Hyalomma excavatum*
LC612455	325	98.15%	KT391040	Israel	*Hyalomma excavatum*
LC612456	325	98.15%	KT391040	Israel	*Hyalomma excavatum*
LC612457	337	98.81%	MH094491	Saudi Arabia	*Hyalomma dromedarii*
LC612458	338	99.70%	KX132904	Niger	*Hyalomma impeltatum*
LC612459	338	99.70%	KX132904	Niger	*Hyalomma impeltatum*
LC612460	338	99.40%	KX132904	Niger	*Hyalomma impeltatum*
LC612461	338	99.40%	KX132904	Niger	*Hyalomma impeltatum*
LC612462	338	99.40%	KX132904	Niger	*Hyalomma impeltatum*
LC612463	338	99.40%	KX132904	Niger	*Hyalomma impeltatum*
LC612464	338	99.70%	KX132904	Niger	*Hyalomma impeltatum*
LC612465	327	100%	MH061014	Greece	*Hyalomma marginatum*
LC612466	327	100%	MH061014	Greece	*Hyalomma marginatum*
LC612467	327	100%	MH061014	Greece	*Hyalomma marginatum*
LC612468	327	100%	MH061014	Greece	*Hyalomma marginatum*
LC612469	327	100%	MH061014	Greece	*Hyalomma marginatum*
LC612433	306	98.37%	KP987776	Israel	*Amblyomma lepidum*
LC612434	306	98.69%	KP987776	Israel	*Amblyomma lepidum*
LC612435	306	98.04%	KP987776	Israel	*Amblyomma lepidum*
LC612436	306	99.02%	KP987776	Israel	*Amblyomma lepidum*
LC612437	306	99.02%	KP987776	Israel	*Amblyomma lepidum*
LC612438	306	99.02%	KP987776	Israel	*Amblyomma lepidum*
LC612439	306	98.69%	KP987776	Israel	*Amblyomma lepidum*
LC612440	349	99.71%	MT906455	Angola	*Amblyomma variegatum*
LC612441	349	100%	MT906455	Angola	*Amblyomma variegatum*

NA: No mitochondrial 12S rDNA reference sequences for *R. simpsoni* are available in the GenBank.
